# Biodegradable Tactile Sensors Using a Bioderived Ionic Liquid for Transient Ionics

**DOI:** 10.1021/acsmaterialsau.5c00107

**Published:** 2025-09-25

**Authors:** Shunsuke Yamada, Muhammad Salman Al Farisi, Momoko Kumemura, Angga Hermawan, Takashi Honda

**Affiliations:** 1 Department of Electrical and Electronic Engineering, 12924 Kyushu Institute of Technology, 1-1 Sensuicho, Tobata, Kitakyushu, Fukuoka 804-8550, Japan; 2 Department of Biomedical Information Sciences, 12801 Hiroshima City University, Hiroshima 731-3194, Japan; 3 Graduate School of Life Science and Systems Engineering, 12924 Kyushu Institute of Technology, 2-4 Hibikino, Wakamatsu ward, Kitakyushu city, Fukuoka 808-0196, Japan; 4 Research Center for Nanotechnology System, National Research and Innovation Agency (BRIN), South Tangerang City, Banten 15314, Indonesia

**Keywords:** ionic liquid, transient electronics, tactile sensor, bioderived materials, biodegradability

## Abstract

Ionic skin is a fundamental platform for tactile sensors that utilize electrolytes as sensing components. Ionic liquids (ILs) are ideal for such applications because of their high ionic conductivities, wide potential windows, and negligible vapor pressures. However, their toxicity hinders their use in wearable, implantable, and environmentally sensitive devices. Herein, ionic gels are synthesized from a bioderived IL and comprise pyramidal microstructures that enhance their tactile sensing capability. These structures improve elasticity and reduce the intrinsic viscoelasticity of the gels. The optimized sensor exhibits two conductance sensitivities of 0.066 and 0.032 and capacitive sensitivities of 0.075 and 0.042 in the pressure ranges of 0–10 kPa and 10–50 kPa, respectively. It exhibits rapid response and relaxation times of 156 and 157 ms, respectively, and maintains sensing capabilities for more than 5000 mechanical cycles, with a change in the conductance of only 8.6%. The sensor degradation test revealed that the active components—the ionic gel and molybdenum (Mo) electrodes—degraded in phosphate-buffered saline within 133 days, whereas the substrate and encapsulation layer remained nondegradable under the tested conditions. These results demonstrate the potential of biodegradable, nontoxic tactile sensors prepared using bioderived ILs in healthcare monitoring, wearable electronics, and environmental sensing.

## Graphical abstract

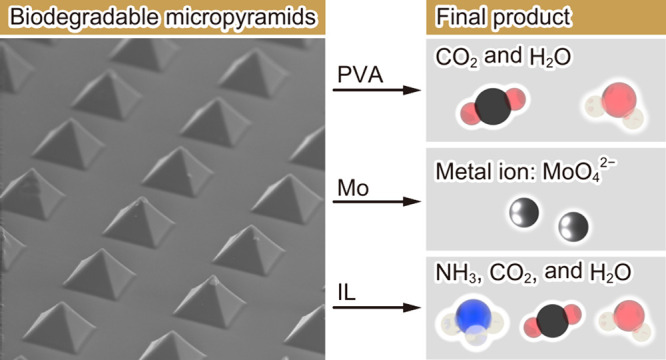

## 1 Introduction

Tactile sensing is crucial for human–machine interfaces in applications such as electronic skins, wearable devices, and robotic manipulation. Materials science has enabled the design of resistive,^1,2^ optical,^3,4^ and capacitive sensors^5–8^ that offer mechanical flexibility and stretchability, allowing conformal integration with soft or curved surfaces, such as skin and prosthetics. Ionic liquids (ILs), composed of organic cations and anions, are attractive for fabricating soft sensing platforms comprising ionic conductors, (ionic skin)^9–11^ because of their high ionic conductivities,^12^ wide electrochemical windows,^13^ and negligible vapor pressures.^14^ ILs can be incorporated into polymer networks to prepare ionic gels (IGs),^15,16^ which form electrical double-layers at electrode interfaces under an applied voltage, enabling high interfacial capacitance (*C*). Micropyramidal features on IG surface can modulate the contact area between an electrode and the IG under pressure, substantially amplifying the changes in the *C* and conductance (*G*) of the sensor.^17,18^ However, conventional IG-based tactile sensors often use toxic ILs such as 1-ethyl-3-methylimidazolium bis­(trifluoromethylsulfonyl)­imide, which limits their application in biomedical and environmental settings.^19^ For applications involving direct human contact or postuse degradation, biocompatible and biodegradable sensing materials are essential. Previous studies have demonstrated tactile sensors fabricated from natural materials such as silk nanofiber,^20^ chitosan and glycerol dielectric layer,^21^ pectin xerogel,^22^ poly­(glycerol sebacate) sheet with pyramid patterns,^23^ and even rose petals.^24^ Those materials exhibited excellent biodegradability attributed to natural resources. Similarly, certain petroleum-derived materials have also shown biodegradability; for example, biodegradable tactile sensors have been developed using polylactic-*co*-glycolic acid (PLGA) and polycaprolactone (PCL) nanofibers.^25^ Devices with such characteristics align with the objectives of transient electronics and are increasingly sought for developing environmentally sustainable sensing technologies.^26–28^

Herein, a biodegradable tactile sensor was developed using a bioderived IL composed of choline and lactate to address the issues associated with the toxicity and degradation of conventional ionic liquids in the environment. Choline and lactate are naturally present in the human body^29–33^ and are considered safe for applications involving direct skin contact, such as wearable devices and electronic skins. Poly­(octamethylene maleate (anhydride) citrate) (EPPOMaC) exhibited excellent biodegradability, biocompatibility, and stretchability. Therefore, we employed EPPOMaC molybdenum, and IG as biodegradable substrates, electrodes, and sensing components, respectively. Moreover, as demonstrated in our previous study, the IL has high biodegradability and can be decomposed by microorganisms, making it a promising candidate for transient electronics.^34,35^ When dispersed in poly­(vinyl alcohol) (PVA), a biodegradable polymer, the ionic liquid forms a biodegradable IG. An IG precursor was cast onto silicon molds featuring reverse-micropyramid patterns with base widths of 25 and 50 μm, yielding IGs with pyramidal structures having rectangular bases. In sensors integrated with these patterns (named T-sensor-25 and T-sensor-50, respectively), T-sensor-50 exhibited high durability under repeated loading, maintaining performance for more than 5000 cycles with a change in *G* of only 8.6%. Multipoint tactile sensing was achieved using an array composed of three T-sensor-50 units, which successfully decoupled pressure signals from neighboring sensors. Degradation tests showed that the Mo electrodes and IG degraded within 133 days, leaving behind small Mo fragments and the EPPOMaC substrate.^36,37^ This study demonstrates the potential of bioderived ILs as biodegradable, transient, and environmentally benign tactile sensing platforms.

## 2 Results and Discussion

### 2.1 Fabrication and Evaluation of IGs

The tactile sensors comprise an EPPOMaC capping layer and substrate, IGs with micropyramidal patterns, and Mo electrodes (Figure 1a). Fabrication began with the preparation of the IGs by dispersing an ionic liquid (IL) into PVA networks, following the procedure reported in our previous study (Figure 1b).^15^ Briefly, a mixture of poly­(vinyl alcohol) (PVA), the IL, and deionized water (DIW) was blade-coated onto a glass substrate at a thickness of 1 mm. After coating, the mixture was dried at ∼25 °C and vacuum-dried to afford a 200-μm-thick IG (Figure 1c). Micropyramidal patterns were formed on the IG surface to modulate the contact area between the IG and the upper molybdenum (Mo) electrodes under applied pressure, resulting in changes in the *C* and *G* (Figure 1d). To investigate the intrinsic electrochemical characteristics of the IG, electrochemical impedance spectroscopy (EIS) measurements were performed on the dried, unpatterned IG. The EIS curves of the IGs resembled those of an electrolyte, containing high and low phase angles at 100 kHz and 0.1 Hz, respectively (Figure 2a). The ionic conductivity (σ) of the IG at 100 kHz was 55 μS cm^–1^, calculated using the following equation:^38–40^

$$\sigma =\frac{t}{AR_{100k}}$$ 1

where *t*, *A*, and *R*_100k_ are the thickness of the IG, the contact area of the Au leaf with the IG, and the resistance of the IG at 100 kHz, respectively. Unless otherwise noted, all values were measured in triplicate and averaged. Herein, the obtained σ was lower than that in our previous studies, where values were approximately 100 μS cm^–1^,^15,34^ because of the higher PVA content used during IG synthesis to prevent IG deformation during the drying process. According to the literature, the IL and PVA form intermolecular interactions via hydrogen bonding, forming supramolecular IGs.^15,41^ Heating supramolecular IGs at temperatures above 100 °C weakens their hydrogen bonding, resulting in thermoplastic behavior that may cause the microstructures on the IG surface to disappear. Therefore, the weight ratio of PVA was increased to preserve the microstructures. The *C* of the IG was calculated using the following equation:^38–40^

$$C=\frac{1}{2\pi f\left|Z_{\mathrm{im}}\right|}$$ 2

where *f* is the input frequency and *Z*_im_ is the imaginary part of impedance. The *C* of the IG was 1.2 μF cm^–2^ at 1 Hz, which is almost the same as that of IGs containing 25 wt % PVA.^15^ The IG surface was slightly wetted due to IL leaching from within. Electrical double-layer capacitance originates due to charge accumulation on the electrode surface upon voltage application. The reduction in the *C* of the IG due to an increase in the PVA ratio was relatively small, especially compared to its effect on the *G* of the IG. This high double-layer capacitance is advantageous for tactile sensing applications, as it enables substantial capacitance modulation in response to applied pressure. Tensile–stress tests showed that the Young’s modulus of the IG was 5.9 MPa, and its fracture stress and strain were 1.1 MPa and 210%, respectively (Figure 2b). When the PVA weight ratio was increased, the IG became more rigid, its electrochemical and mechanical proper ties were altered, and its transparency meanwhile remained high (>90%) in the wavelength range of 200–1000 nm (Figure 2c). Microstructures were fabricated on the IG surface using Si molds featuring reverse-pyramid patterns with square bases of 25 μm × 25 or 50 μm × 50 μm. The IG precursor was cast onto Si molds and blade-coated with a thickness of 1 mm above the mold surface, followed by drying for 24 h. After gelation, the IGs were peeled from the molds (Figure 2d) and vacuum-dried at 50 °C for 24 h. The areas on the IGs with pyramid patterns appeared translucent white. We evaluated the optical transmittance of IG60 samples with and without pyramid patterns. The unpatterned IG60 exhibited an average transmittance of approximately 90% in the visible range (380–780 nm) (Figure S1, Supporting Information). In contrast, patterned IG60 samples showed significantly reduced transmittance due to light scattering caused by the surface microstructures. Specifically, gels with 25 and 50 μm-base pyramidal patterns exhibited average transmittance values of 56% and 37%, respectively. The decrease in transmittance is attributed to enhanced scattering from larger pyramid structures. Scanning electron microscopy confirmed the successful transfer of pyramid patterns onto the IGs. Due to shrinkage caused by water evaporation, the sizes of the pyramid patterns prepared using 25 μm × 25 and 50 μm × 50 μm molds decreased to 20 μm × 20 and 40 μm × 40 μm, respectively (Figure 2e,f).

**Figure 1:**
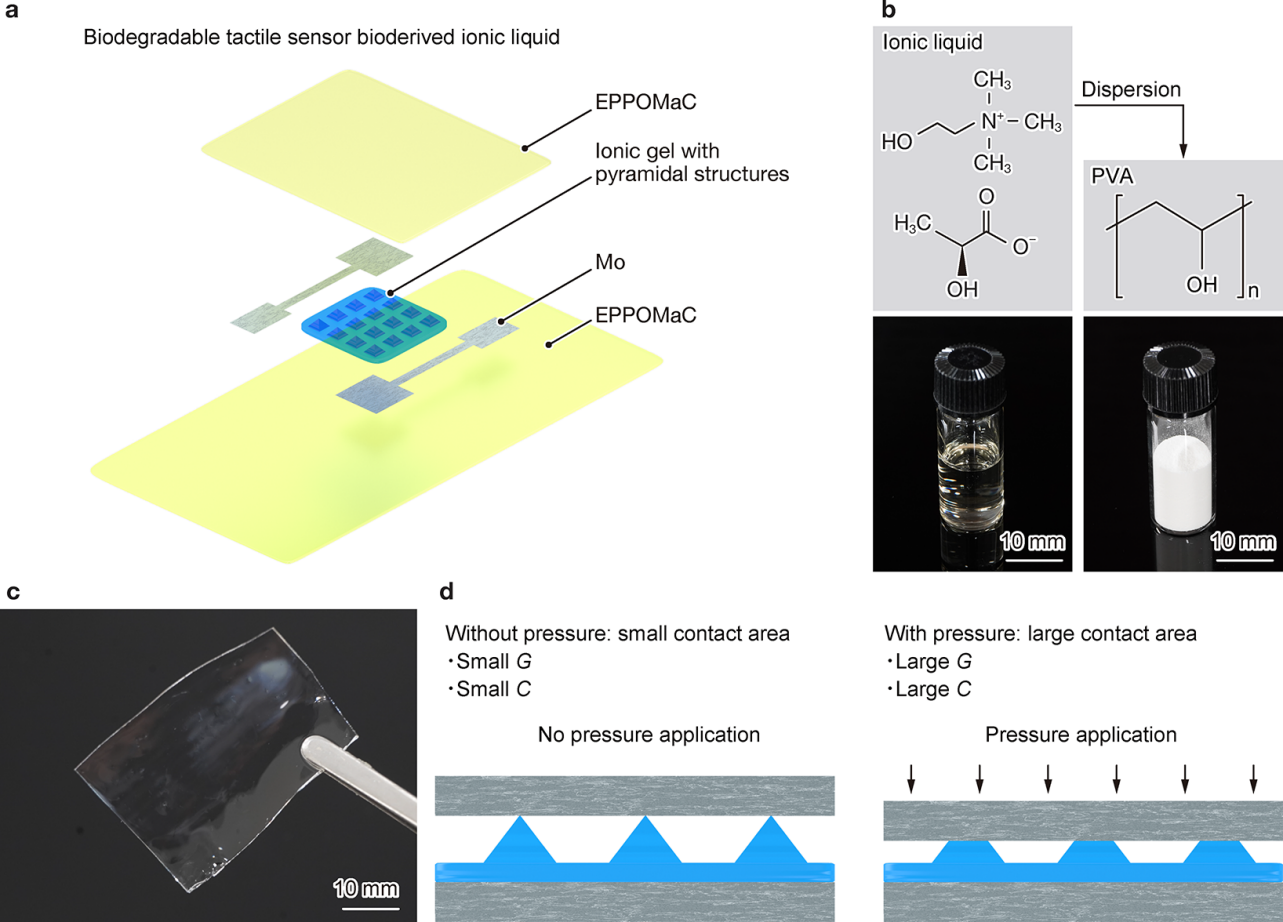
Schematic illustration and sensing mechanism of the tactile sensor. (a) Schematic of the sensor composed of Mo foils, an IG with pyramid structures, EPPOMaC electrodes, a sensing component, and substrates. (b) Chemical formula and photograph of PVA and the IL. (c) Dispersing the IL into PVA forms a freestanding IG. (d) Pressure application deforms the pyramid structures on IGs, changing the contact area between the Mo electrode and IG, which increases the ionic conductance *G* and capacitance *C* of the sensor.

**Figure 2:**
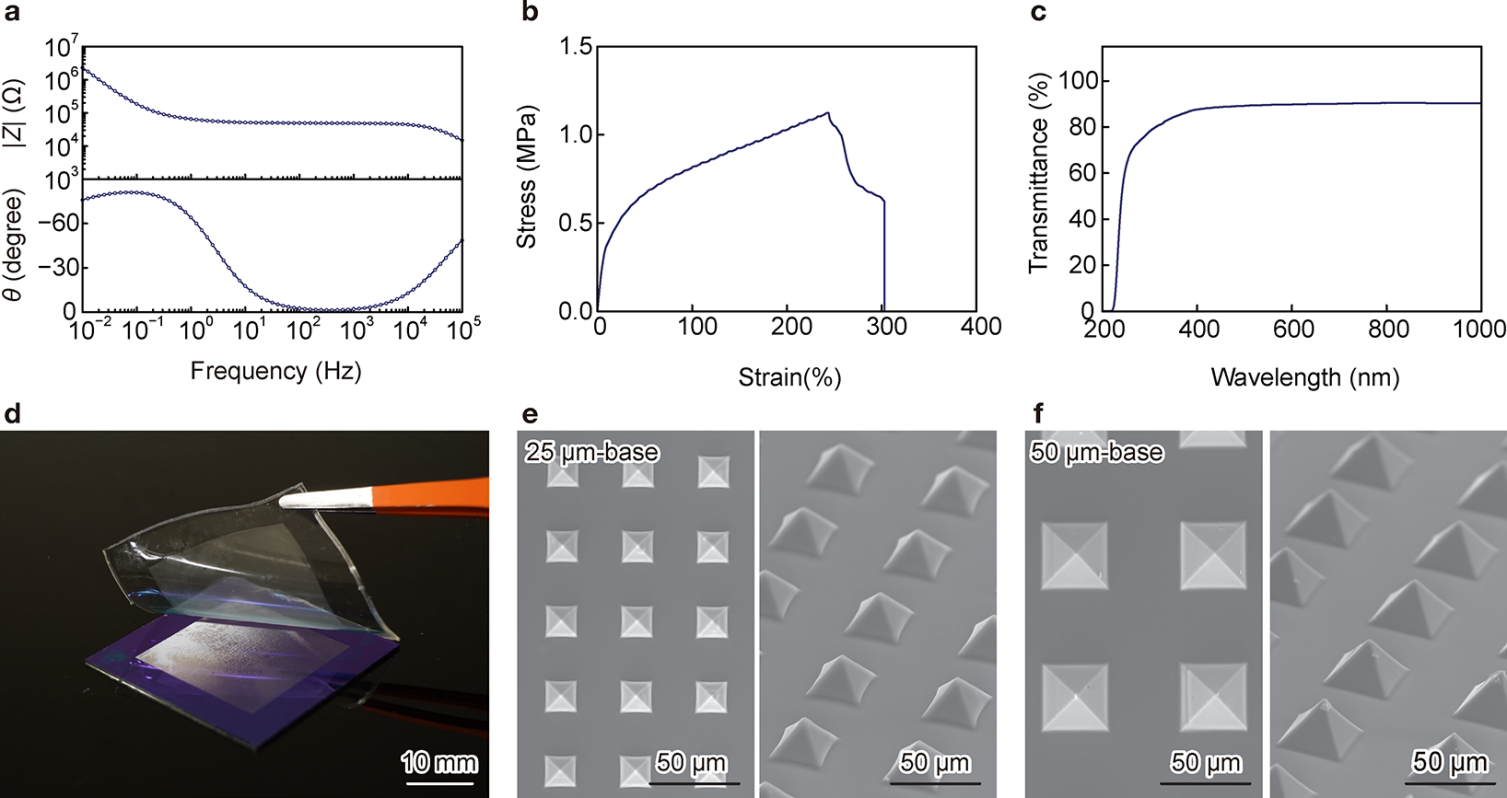
Characterization of IG60 and transfer of pyramidal structures. (a) EIS spectrum, impedance, and phase. (b) Strain–stress curve. (c) Transmittance of IG60 obtained via UV–vis spectroscopy. (d) Transfer of the pyramidal structures onto IG60 using a Si mold. (e, f) SEM image of the developed pyramidal structures using molds with (e) 25 or (f) 50 μm-base reverse-pyramidal structures.

### 2.2 Fabrication and Characterization of Tactile Sensors

Tactile sensors were fabricated using IGs with pyramidal structures (Figure 3a). EPPOMaC was synthesized according to the procedure reported by Tran et al. and Boutry et al. with some modifications.^36,37^ An EPPOMaC sheet with dimensions of 20 mm × 40 mm × 1 mm was used as the substrate and capping layers. A Mo electrode with a surface area of 5 mm × 5 mm was placed on the EPPOMaC sheet, and an IG (7 mm × 7 mm) was laminated onto the electrode. Another Mo electrode was placed on top of the IG to prepare the Mo–IG–Mo configuration, which was encapsulated with an EPPOMaC sheet. The changes in the *G* and *C* of the sensor under applied pressure were evaluated. A sensor composed of an IG without pyramid patterns was used as the control. Hereafter, the tactile sensor without pyramid patterns is called T-sensor-0. The change in the *G* of the sensor as a function of the applied static pressure (*P*) is evaluated using the following equation:

$$\Delta G=\frac{G-G_{0}}{G_{0}}$$ 3

where *G*_0_ is the *G* of the sensor without applied pressure. The Δ*G* values of T-sensor-25 and T-sensor-50 increased with increasing pressure (Figure 3b) whereas those of T-sensor-0 exhibited a negligible change. The sensitivity (*S*_*G*_) of the sensor to Δ*G* is defined as follows:^17^

$$S_{G}=\frac{\Delta G}{\Delta P}$$ 4

**Figure 3:**
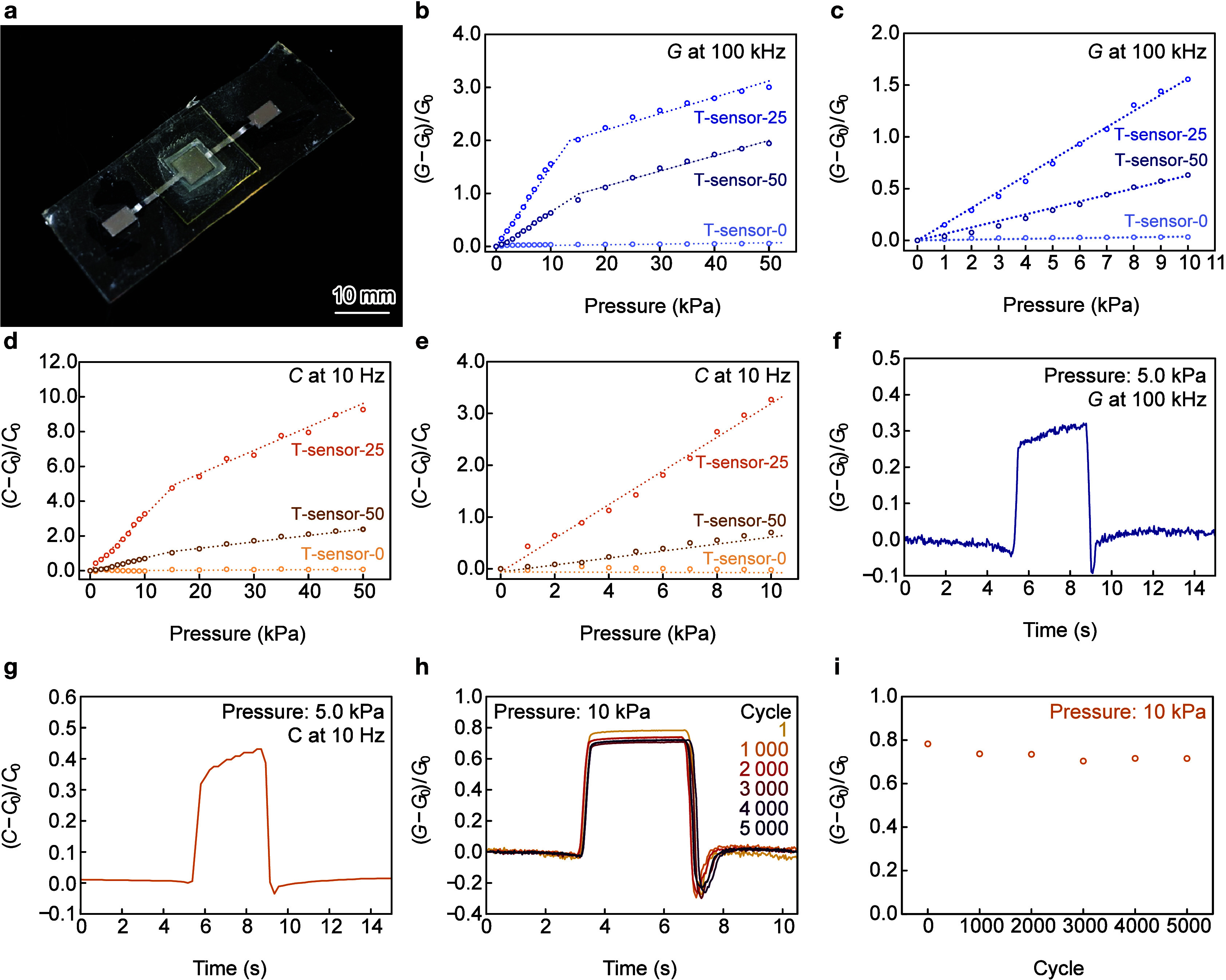
Characterization of the tactile sensors. (a) Photograph of the developed T-sensor-50 sensor. Change in the ionic conductivities of T-sensor-0, T-sensor-25, and T-sensor-50 as a function of the applied pressure in the pressure ranges of (b) 0–50 kPa and (c) 0–10 kPa. Change in the capacitances of T-sensor-0, T-sensor-25, and T-sensor-50 as a function of the applied pressure in the pressure ranges of (d) 0–50 kPa and (e) 0–10 kPa. (f, g) Response and relaxation behavior of T-sensor-50 in terms of its ionic conductance and capacitance. (h) Response and relaxation behavior of T-sensor-50 in terms of its conductance during a 5000-cycle stability test. (i) Change in the conductance of T-sensor-50 during the 5000-cycle stability test.

The Δ*G* plots of T-sensor-25 and T-sensor-50 exhibited two distinct *S*_*G*_ values in the pressure ranges of 0–10 kPa and 10–50 kPa. T-sensor-25 exhibited high and low sensitivities of 0.16 and 0.033 kPa^–1^, with R-squared values of 0.997 and 0.975 in the 0–10 kPa and 10–50 kPa ranges, respectively. In the same pressure ranges, T-sensor-50 exhibited *S*_*G*_ values of 0.066 and 0.032 kPa^–1^, with R-squared values of 0.993 and 0.979, respectively. The high sensitivities arise from the significant increase in contact area between the electrode and the IG. Upon applying pressures above 10 kPa, the deformation of the micropyramidal structures diminishes, resulting in reduced sensitivity. Magnified plots in the pressure range of 0–10 kPa revealed that T-sensor-25 exhibited a higher *S*_*G*_ value than T-sensor-50 (Figure 3c). The *C* of T-sensor-25 and T-sensor-50 increased with increasing *P* due to the increase in the electrical double-layer capacitance caused by the increased contact area between the electrodes and IGs. The change in the *C* (Δ*C*) of the sensor is calculated as follows:

$$\Delta C=\frac{C-C_{0}}{C_{0}}$$ 5

where *C*_0_ is the *C* of the sensor without applied pressure. The sensitivity of capacitance (*S*_C_) is defined as follows:^17^

$$S_{C}=\frac{\Delta C}{\Delta P}$$ 6

The Δ*C* plots of T-sensor-25 and T-sensor-50 also exhibited two distinct *S*_C_ values in the pressure ranges of 0–10 kPa and 10–50 kPa (Figure 3d). In the pressure ranges of 0–10 and 10–50 kPa, T-sensor-25 exhibited *S*_C_ values of 0.32 and 0.14 kPa^–1^ with R-squared values of 0.991 and 0.983, respectively, whereas T-sensor-50 showed values of 0.075 and 0.042 kPa^–1^ with R-squared values of 0.988 and 0.985 (Figure 3e). These results indicate that 25-μm-base pyramids deformed more easily under applied pressure than 50-μm-base pyramids, increasing the contact area with th e Mo electrode, yielding high *S*_*G*_ and *S*_C_ values in the 10–50 kPa pressure range.

Rapid response and relaxation times are essential for tactile sensing applications. When a pressure of 5.0 kPa was applied to T-sensor-50, its Δ*G* increased to 63% of steady state within 156 ms (Figure 3f). Upon release of the pressure, T-sensor-50 exhibited a relaxation time of 157 ms to reach 37% of steady state. For Δ*C*, the response and relaxation times were 408 and 422 ms (Figure 3g), respectively. Previous studies on tactile sensors have reported that microstructures on sensing components impart elasticity, shortening their relaxation time. Mannsfeld et al. reported that a tactile sensor incorporating a viscoelastic material (i.e., polydimethylsiloxane (PDMS)) exhibited a relaxation time of >10 s due to the irreversible entanglement of polymer chains.^18^ Similarly, Cho et al. demonstrated that a tactile sensor prepared using an unpatterned IG exhibited a long relaxation time because of the viscoelastic and adhesive nature of the IG, particularly when loaded with over 50% IL.^17^ Incorporating a pyramidal structure on the gel surface enables response and relaxation to occur within 20 ms. Herein, pyramidal structures effectively minimized the viscoelastic behavior of the IGs, resulting in rapid response and relaxation times. By contrast, T-sensor-25 exhibited response and relaxation times of 108 ms and 23 s for Δ*G* (Figure S2a, Supporting Information). This difference between the response and relaxation times may be attributed to the friction between EPPOMaC layers. The sensor contained air pockets between the EPPOMaC capping layer and substrate. Upon pressure-induced deformation, the EPPOMaC layers adhered to each other due to the sticky nature of EPPOMaC. As a result, a repulsive force was required to separate these layers after deformation.

The 25-μm-base pyramids provided insufficient repulsive force, resulting in a prolonged relaxation time or hysteresis. Consequently, the Δ*C* signal did not return to 0 after pressure release (Figure S2b), further indicating hysteresis caused by pressure-induced adhesion. Although T-sensor-25 exhibited high *S*_*G*_ and *S*_C_ values, its prolonged relaxation time for Δ*G* and hysteresis in Δ*C* were not favorable for tactile sensing applications. Therefore, the stability of T-sensor-50 was studied using cyclic pressure loading and unloading. Its Δ*G* response remained unchanged during the first, 1000th, 2000th, 3000th, 4000th, and 5000th cycles, during which a stress tester applied a pressure of 10 kPa for 3 s. As the number of cycles increased, the peak Δ*G* values of T-sensor-50 slightly decreased (Figure 3h); however, it exhibited consistent response and relaxation times. The peak Δ*G* value of T-sensor-50 was 8.6% lower during the 5000th cycle than the first cycle (Figure 3i). Despite this minor degradation, T-sensor-50 retained its sensing capability after 5000 cycles, demonstrating high mechanical robustness for tactile sensing applications. Compared to previous tactile sensors, T-sensor-50 exhibited excellent sensitivity over a pressure range of 0–50 kPa, a fast response time, and high cyclic durability (Table S1, Supporting Information).

### 2.3 Tactile Sensor Array and Its Demonstration

We fabricated an array of sensors for multiple tactile detection using three T-sensor-50 units (Figure 4a). The array was prepared by sequentially laminating Mo electrodes, IGs, and Mo electrodes on an EPPOMaC sheet, which was sealed with a capping EPPOMaC layer. When a finger individually pressed one sensor, only the *G* of that sensor changed, indicating that the sensors were decoupled from pressure applied to neighboring units (Figure S3). While the middle T-sensor-50 exhibited Δ*G* of approximately 0 without applied pressure, the left and right T-sensor-50 exhibited Δ*G* values of 0.31 and 0.22, respectively, when pressure was applied with two fingers (Figure 4b,c). The middle T-sensor-50 did not respond to finger presses, indicating that the array was capable of multipoint sensing. The response of the middle T-sensor-50 was demonstrated by recording a message in Morse code (Figure 4d). Figure 4e shows the response of T-sensor-50 to finger presses, where short and long presses denoted a dot and a dash, respectively. The signal of Δ*G* increased when the pressure was applied with a finger and returned to 0 after the pressure was removed, indicating its quick response and relaxation times. Variation in the absolute Δ*G* peaks was observed due to fluctuations in the pressure applied manually by a human finger during testing. Nevertheless, the response and stability of the sensor made dashes and dots distinguishable and reliable, and the signal from T-sensor-50 was decoded as “IONIC LIQUID.” These results demonstrate the suitability of T-sensor-50 for applications in tactile sensing, wearable devices, i-skin, and displays.

**Figure 4:**
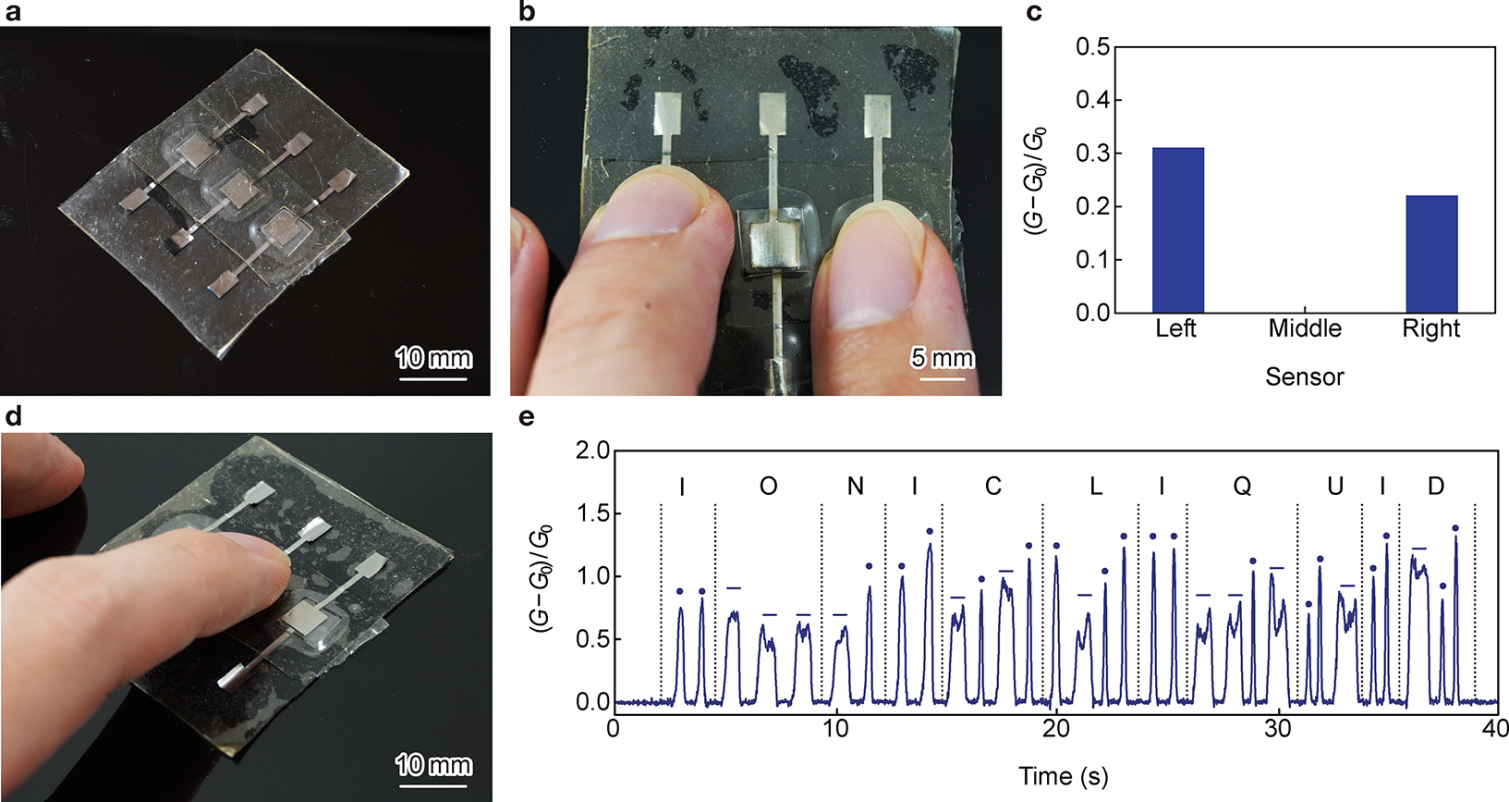
Characterization of the array containing multiple T-sensor-50 units. (a) Photograph of an array containing three T-sensor-50 units. (b) Pressure application on the left and right T-sensor-50 units. (c) Corresponding change in *G* indicates decoupled detection with individual sensors. (d) Detection of Morse codes using the middle T-sensor-50. (e) Short and long pressing of the T-sensor-50 corresponded with dashes and dots, respectively, and the signal was decoded as “IONIC LIQUID.”

### 2.4 Degradation Test of the Tactile Sensor

Biodegradability is essential to realize eco-friendly sensors that disappears with nature after passing its lifetime. We investigated the transient behavior of T-sensor-50 without a capping layer after soaking it in 100 mL of a phosphate-buffered saline (PBS) solution stored in an oven at 37 °C. The Mo foils were a glossy gray color on day 1 and turned a dark gray color by day 63 due to oxidation (Figure 5a). The IG dissolved into the PBS by day 63. The hydrolysis of Mo oxide decreased the thickness of Mo foils, and wrinkles and cracks appeared on Mo foils by day 119. One Mo foil broke into pieces, and the other almost disappeared, leaving only a thin Mo oxide layer by day 133. The change in the mass (Δ*M*) of the sensor is described as follows:

$$\Delta M=\frac{M-M_{0}}{M_{0}}$$ 7

where *M* and *M*_0_ are the masses of the sensor during the degradation test and on the first day of the test, respectively. The Δ*M* of T-sensor-50 rapidly decreased within the first 20 days due to IG dissolution, followed by a linear decrease (Figure 5b). The EPPOMaC substrate did not dissolve in PBS, but its texture became rigid after soaking, which contributed to the remaining mass after day 133. EPPOMaC comprised photo-cross-linked monomers, and this chemically stable bonding prevented EPPOMaC from dissolving in the PBS solution. The micropyramidal structure may undergo changes during the degradation test. In future work, we will systematically characterize the morphological evolution of these features using three-dimensional profilometry to elucidate structure–performance relationships. As water-soluble substrates, poly­(1,8-octanediol-*co*-citrate), PVA, and poly­(glycerol sebacate) are promising candidates for sensor substrates because of their hydrolysis and polymer/monomer biodegradability.

**Figure 5:**
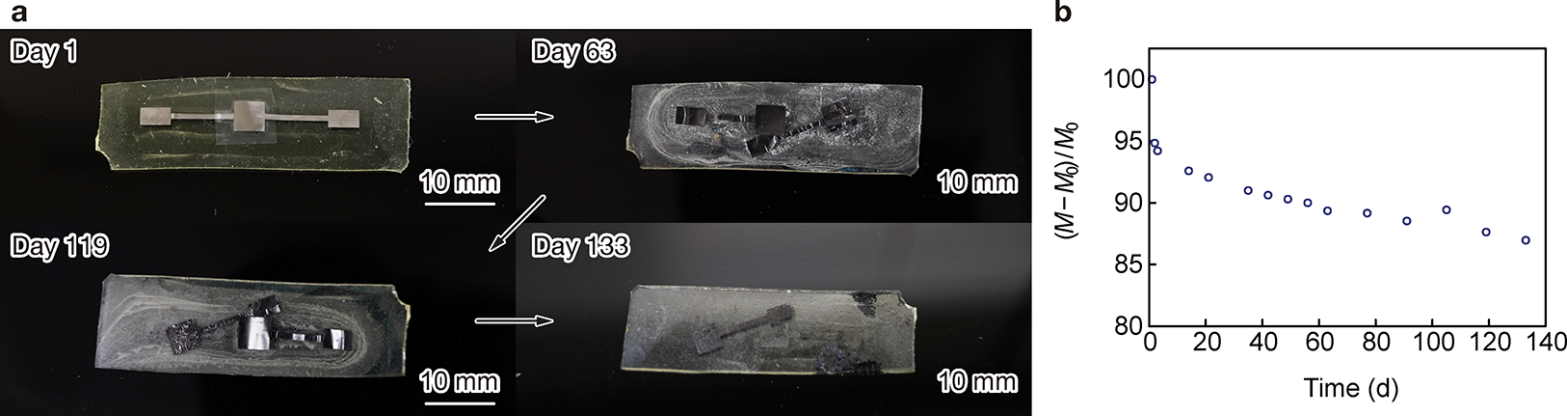
Degradation test of T-sensor-50. (a) Change in the appearance and (b) corresponding mass of T-sensor-50. T-sensor-50 was stored in 10 mM PBS at 37 °C.

## 3 Conclusions

We developed tactile sensors using bioderived IGs with pyramidal structures. The pyramidal structures created air gaps between the IG and Mo electrode in the absence of applied pressure, maintaining their small contact area. Upon applying pressure, the pyramidal structures deformed to increase the contact area, increasing the *C* and *G* of the sensor. The optimized sensor T-sensor-50 exhibited two sensitivities of 0.066 and 0.032 in the pressure ranges of 0–10 kPa and 10–50 kPa, respectively. Its capacitive sensitivities were 0.075 and 0.042 in the same ranges, respectively. The pyramidal structures imparted elasticity and reduced the inherent viscoelastic behavior of the IGs. Consequently, T-sensor-50 exhibited response and relaxation times of 156 and 157 ms, respectively, upon the application and release of pressure. Degradation testing of the T-sensor-50 demonstrated that the ionic gel and Mo electrodes underwent degradation in phosphate-buffered saline within 133 days, while the EPPOMaC substrate and encapsulation layer remained stable under the same conditions. This study demonstrated the material synthesis, device fabrication, and characterization of a biodegradable i-skin prepared using bioderived materials, expanding its applications to healthcare, wearable, and environmental sensing.

## 4 Experimental Section

### 4.1 Synthesis of the IL and Gel

Choline bicarbonate (product ID: C7519, Merck) was slowly added to lactic acid (product ID: 27715, Merck) in a molar ratio of 1:1 and stirred for 24 h with a magnetic stirrer. Next, water was removed from the resulting solution using a rotary evaporator at 50 °C and ∼5 kPa until bubbling ceased. The solution was decanted into a flask and vacuum-dried at 50 °C and ∼10 Pa for 24 h to remove the residual moisture. Next, the IL, PVA, and DIW were mixed in a weight ratio of 3:2:12. The mixture was transferred into a glass bottle and heated at 110 °C to obtain a clear solution. The solution was blade-coated onto a glass plate using a thickness of 1 mm and left to gelatinize under ambient conditions (∼24 h). The resulting IG was cut and vacuum-dried at 50 °C and 5 Pa for 24 h to remove water before use, yielding a 200-μm thickness.

### 4.2 Si Mold Fabrication

To fabricate the Si mold, 4-in. silicon wafers (n-type, 1–20 Ω·cm, < 100> orientation, thickness = 300 μm) were oxidized to passivate their surfaces with a 300 nm-thick silicon dioxide (SiO_2_) layer. The wafers were first diced into squares with dimensions of 30 mm × 30 mm using a blade dicer (DAD3240, DISCO) and then cleaned with a piranha solution composed of 95% H_2_SO_4_ + 30% H_2_O_2_ in a volume ratio of 1:2. After spin-coating the photoresist (OFPR 800LB, 200 cP, TOKYO OHKA KOGYO CO., Ltd.) onto Si substrates, photolithography and development were performed to create square window patterns on each substrate. The first substrate had patterns with dimensions of 25 μm × 25 μm and a spacing of 25 μm, and the second substrate had patterns with dimensions of 50 μm × 50 μm and a spacing of 50 μm, each covering an area of 20 mm × 20 mm. An inductively coupled plasma reactive ion etching (ICP-RIE) system (CE-300I, ULVAC Technologies, Inc.) was used to etch the SiO_2_ layer and expose the Si surface. The photoresist was stripped using a remover (MS2001, FUJIFILM Electronic Materials Co., Ltd.), and the residual resist was further removed via piranha solution treatment. Then, reverse-pyramidal structures were formed on Si substrates via anisotropic etching using a tetramethylammonium hydroxide (TMAH) solution (Tama Chemicals Co., Ltd.) at 80 °C. The Si molds were cleaned with the piranha solution before use to render their surface hydrophilic, allowing it to be uniformly coated with the IG precursor. The IG precursor was blade-coated onto the Si molds using a blade coater with a thickness of 1 mm from the mold surface. The coated IGs were stored at ∼25 °C for 24 h to allow gelatinization. After gelatinization, the IGs were peeled off from the Si molds, cut, and vacuum-dried at 50 °C for 24 h before use.

### 4.3 Device Fabrication and Characterization

A laser processing machine (ProtoLaser U4, LPKF Laser & Electronics KK) was used to cut a 10-μm-thick Mo foil, which was soaked in a 29% ammonia solution to remove native oxide layers. Two patterned Mo foils and an IG with pyramidal structures having base widths of 25 or 50 μm were transferred onto an EPPOMaC substrate to prepare the Mo–IG–Mo configuration. An EPPOMaC precursor served as an adhesive to attach the EPPOMaC capping layer to the EPPOMaC substrate supporting the Mo foils and IG. The sensor was heated at 100 °C for 6 h to complete polymerization, yielding encapsulated tactile sensors. A tensile–stress tester applied pressure to the tactile sensors through a silicone rubber sheet (10 × 10 mm), during which the sensors were characterized using an EIS module (FRA32M, Autolab), and their impedance response was measured with an impedance analyzer (IM3590, HIOKI).

### 4.4 Degradation Test

A 10 mM PBS solution was prepared by dissolving NaCl (8.0 g), KCl (0.2 g), Na_2_HPO_4_·12H_2_O (2.9 g), and KH_2_PO_4_ (2.9 g) in 1 L of DIW. T-sensor-50 was immersed in 100 mL of the PBS solution, which was maintained at 37 °C in an oven. The solution was replaced daily. T-sensor-50 was vacuum-dried at 40 °C for 3 h before weight measurement.

## Supplementary Material



## Notes

Figures S1–S3: Optical transparency comparison of IG60 with and without micropyramidal patterns, response of T-sensor-25 in conductance and capacitance modes, conductance changes in the T-sensor-50 array in response to individual poking; Table S1: comparison of tactile sensor performance in recent literature (PDF)
