# Supplementary material for: Biodegradable Tactile Sensors Using a Bioderived Ionic Liquid for Transient Ionics
Source: ACS Mater Au. 2025 Sep 25;5(6):1029–36. doi: 10.1021/acsmaterialsau.5c00107 (PMC12616432; doi:10.1021/acsmaterialsau.5c00107)
Supplement: Supplementary file 1 [file mg5c00107_si_001.pdf]

Supporting Information

Biodegradable Tactile Sensors Using a Bioderived Ionic Liquid for Transient Ionics

Shunsuke Yamada[1]*, Muhammad Salman Al Farisi[2], Momoko Kumemura[3], Angga Hermawan[4] and Takashi Honda[1]

[1]Department of Electrical and Electronic Engineering, Kyushu Institute of Technology, 1-1 Sensuicho, Tobata, Kitakyushu, Fukuoka 804-8550, Japan

[2]Department of Biomedical Information Sciences, Hiroshima City University, Hiroshima 731-3194, Japan

[3]Graduate School of Life Science and Systems Engineering, Kyushu Institute of Technology, 2-4 Hibikino, Wakamatsu ward, Kitakyushu city, Fukuoka 808-0196, Japan;

[4]Research Center for Nanotechnology System, National Research and Innovation Agency (BRIN), South Tangerang City, Banten 15314, Indonesia

Corresponding Author
Shunsuke Yamada (orcid.org/0000-0002-9084-2070)
e-mail: yamada@ele.kyutech.ac.jp

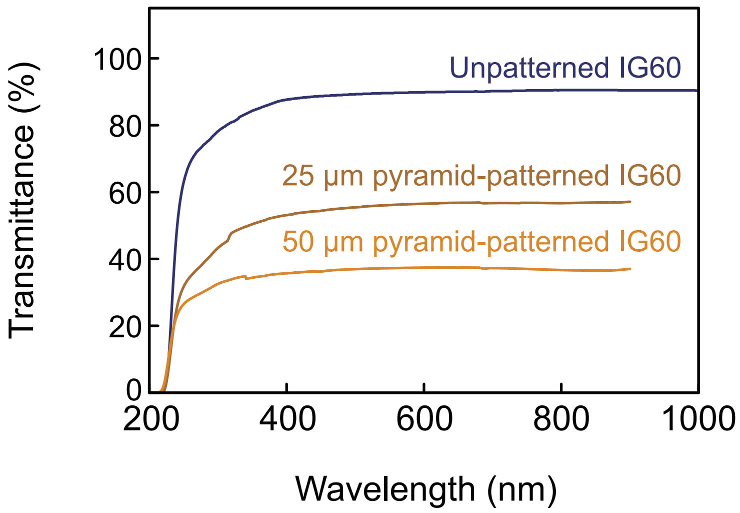

Figure S1. Optical transmittance of IG60 with and without pyramidal surface patterns. The presence of pyramid patterns increases light scattering, resulting in reduced transmittance. Transmittance decreases further as the base width of the pyramids increases.

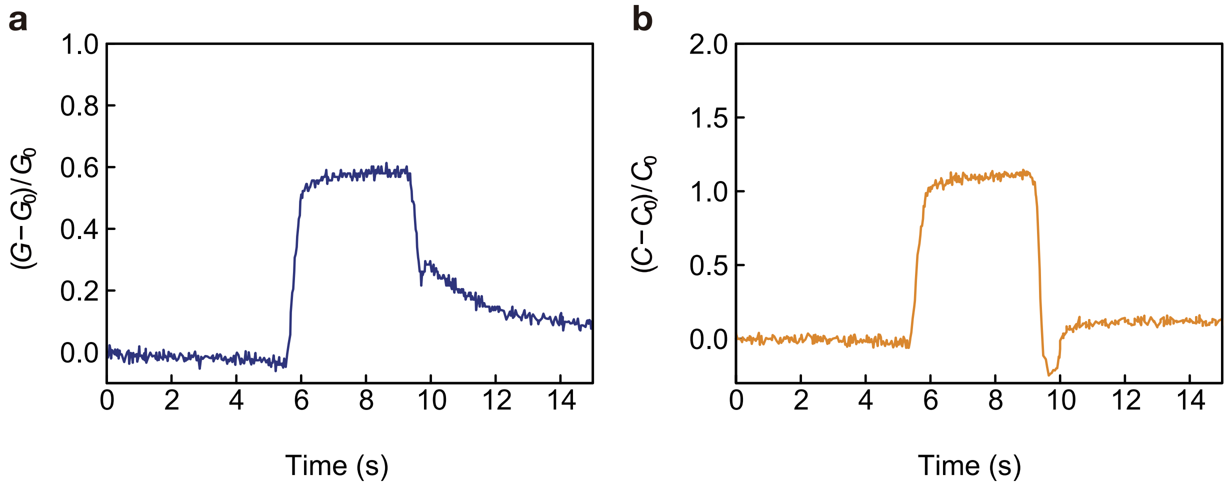

Figure S2. Characterization of the T-sensor-25. Response and relaxation behavior of T-sensor-25 in terms of (a) conductance and (b) capacitance, respectively.

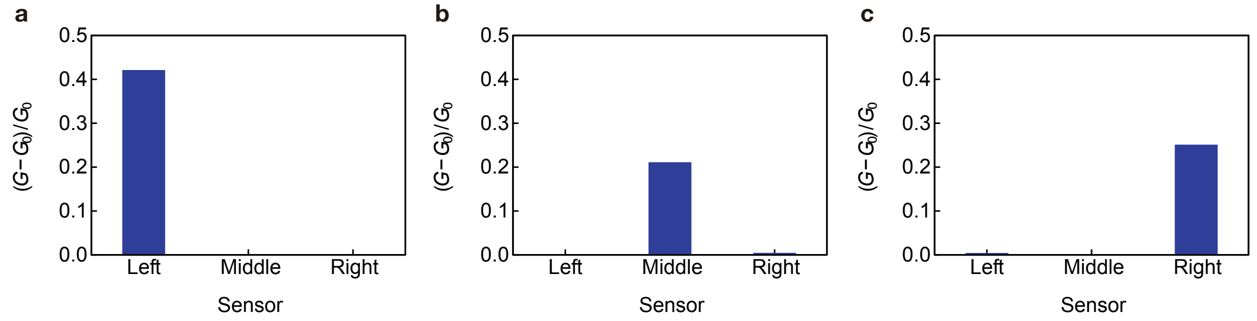

Figure S3. Characterization of T-sensor-50 array. Variation in conductance for (a) left, (b) middle, and (c) right sensors respectively, induced by poking individual sensors.

Table S1. Comparison of sensing materials, sensing area, response time, thermal index, thermal activation energy, temperature coefficient of resistance, sensing resolution, and aging test from recent studies on temperature sensors.

Sensing material	Sensitivity (kPa^{-1})	Pressure range	Response time (ms)	Cyclic number	Year	Ref.
Ionic gel ([Ch][Lac])	Conductance:<br>0.066 (0–10 kPa)<br>0.032 (10–50 kPa)	0–50 kPa	156 ms	5000	2025	This work
Pectin xerogel	0.0294	0.1–1 kPa	118 ms	10000	2024	1
Glycerol/chitosan	1.51 (0–0.3 kPa)	0–40 kPa	180 ms	1700	2022	2
Rose petal	0.08	0.007–60 kPa	-	5000	2020	3
Poly(glycerol sebacate)	0.863 ± 0.025 (0–1.86 kPa)<br>0.062 ± 0.005 (1.86–4.6 kPa)	0-4.6 kPa	251 ms	2200	2019	4
Ecoflex	0.01887 (<45 kPa)<br>5.5037×10^{-4} (>300 kPa)	35 Pa–700 kPa	-	7200 (compression)	2019	5
PLGA and PCL nanofiber	0.76 ± 0.14 (0–2 kPa)<br>0.11 ± 0.07 (2–10 kPa)	0–10 kPa	Millisecond range	8000	2015	6

Reference

1. Basarir, F.; Haj, Y. A.; Zou, F.; De, S.; Nguyen, A.; Frey, A.; Haider, I.; Sariola, V.; Vapaavuori, J., Edible and biodegradable wearable capacitive pressure sensors: a paradigm shift toward sustainable electronics with bio-based materials. *Advanced Functional Materials* **2024,** *34* (39), 2403268.
2. Song, Z.; Liu, Z.; Zhao, L.; Chang, C.; An, W.; Zheng, H.; Yu, S., Biodegradable and flexible capacitive pressure sensor for electronic skins. *Organic Electronics* **2022,** *106*, 106539.
3. Elsayes, A.; Sharma, V.; Yiannacou, K.; Koivikko, A.; Rasheed, A.; Sariola, V., Plant-based biodegradable capacitive tactile pressure sensor using flexible and transparent leaf skeletons as electrodes and flower petal as dielectric layer. *Advanced Sustainable Systems* **2020,** *4* (9), 2000056.
4. Khalid, M. A. U.; Ali, M.; Soomro, A. M.; Kim, S. W.; Kim, H. B.; Lee, B.-G.; Choi, K. H., A highly sensitive biodegradable pressure sensor based on nanofibrous dielectric. *Sensors and Actuators A: Physical* **2019,** *294*, 140-147.
5. Hou, C.; Xu, Z.; Qiu, W.; Wu, R.; Wang, Y.; Xu, Q.; Liu, X. Y.; Guo, W., A biodegradable and stretchable protein-based sensor as artificial electronic skin for human motion detection. *Small* **2019,** *15* (11), 1805084.
6. Boutry, C. M.; Nguyen, A.; Lawal, Q. O.; Chortos, A.; Rondeau-Gagné, S.; Bao, Z., A sensitive and biodegradable pressure sensor array for cardiovascular monitoring. *Advanced Materials (Deerfield Beach, Fla.)* **2015,** *27* (43), 6954-6961.
